# Muscular Cystic Lesions: A Highly Misdiagnosed Extraosseous Ewing Sarcoma: Two Case Reports and Literature Review

**DOI:** 10.2174/0115734056353279250311052757

**Published:** 2025-04-29

**Authors:** Deng Xiang, Hui Huang, Xiaozhen Meng, Yun Hu, Shouhua Zhang

**Affiliations:** 1 Department of General Surgery, The Affiliated Children’s Hospital of Nanchang Medical College, Jiangxi Provincial Children’s Hospital, Nanchang 330006, Jiangxi, China; 2 Department of Pathology, The Affiliated Children’s Hospital of Nanchang Medical College, Jiangxi Provincial Children’s Hospital, Nanchang 330006, Jiangxi, China; 3 Jiangxi Medical College, Nanchang University, Nanchang 330006, Jiangxi, China

**Keywords:** Ewing Sarcoma, Color Doppler Flow Imaging, Fluorescence in situ hybridization, Computed Tomography, Muscular Cystic Lesions, Lymphatic malformations

## Abstract

**Background:**

A retrospective analysis was carried out on two cases of extraosseous Ewing sarcoma (ES) that were initially misdiagnosed as lymphatic malformations, with a focus on clinical manifestations, imaging characteristics, and other relevant case data. A comprehensive review of the literature was performed to enhance the understanding of cystic extraosseous ES.

**Case Presentation:**

Both cases in this study originated from cystic lesions in the muscular interstitial space. Due to the absence of distinctive clinical manifestations and imaging features, the diagnosis is primarily dependent on pathological examination.

**Conclusion:**

It is crucial to differentiate this condition from lymphatic malformations, hemangiomas, hematomas, and other diseases to ensure accurate diagnosis and appropriate treatment.

## 1 INTRODUCTION

Ewing Sarcoma (ES) is a highly malignant small round cell tumor, comprising approximately 10% of all primary malignant bone tumors, mainly originating in the long bones [1, 2]. Rarely, it can occur in mesenchymal tissues and other locations, referred as Extraskeletal Ewing Sarcoma (EES). EES is a highly aggressive, rapidly progressive, and metastatic tumor, mainly affecting adolescents aged 10-30, with extremely rare cases in young children. ES shows a slight male predominance, with a male-to-female ratio of 3:2 [3]. EES can occur in virtually any organ, typically in deep soft tissues, and is most commonly found in the paraspinal region, lower limbs, chest wall, retroperitoneum, pelvis, and buttocks [4]. ES of the head and neck is extremely rare [5], accounting for less than 1% to 4% of all cases [1, 2]. Studies have reported that ES is associated with chromosomal translocations involving the EWSR1 gene on chromosome 22q12 [6]. This study presents the diagnosis and treatment of two pediatric patients admitted to our hospital by analyzing the causes of misdiagnosis to en-hance understanding of this disease. This study was approved by the ethics committee of Jiangxi Provincial Child-ren’s Hospital (JXSETYY-YXKY-20220068). Informed con-sent was obtained from the parents or guardians of all participants.

## 2 CASE DESCRIPTION

### 2.1 Case 1

A 1-year-old girl was admitted to the hospital with a 20-day history of a mass in the right buttock. The patient had no other symptoms and was in good general condition with no significant past medical history.

#### 2.1.1 Physical Examination

A protruding mass was visible in the right buttock, with intact skin and no ulceration or fluid discharge. The mass was tense, firm in texture, had well-defined margins, was slightly tender, and exhibited some mobility.

#### 2.1.2 Ultrasound

**Fig. (1):**
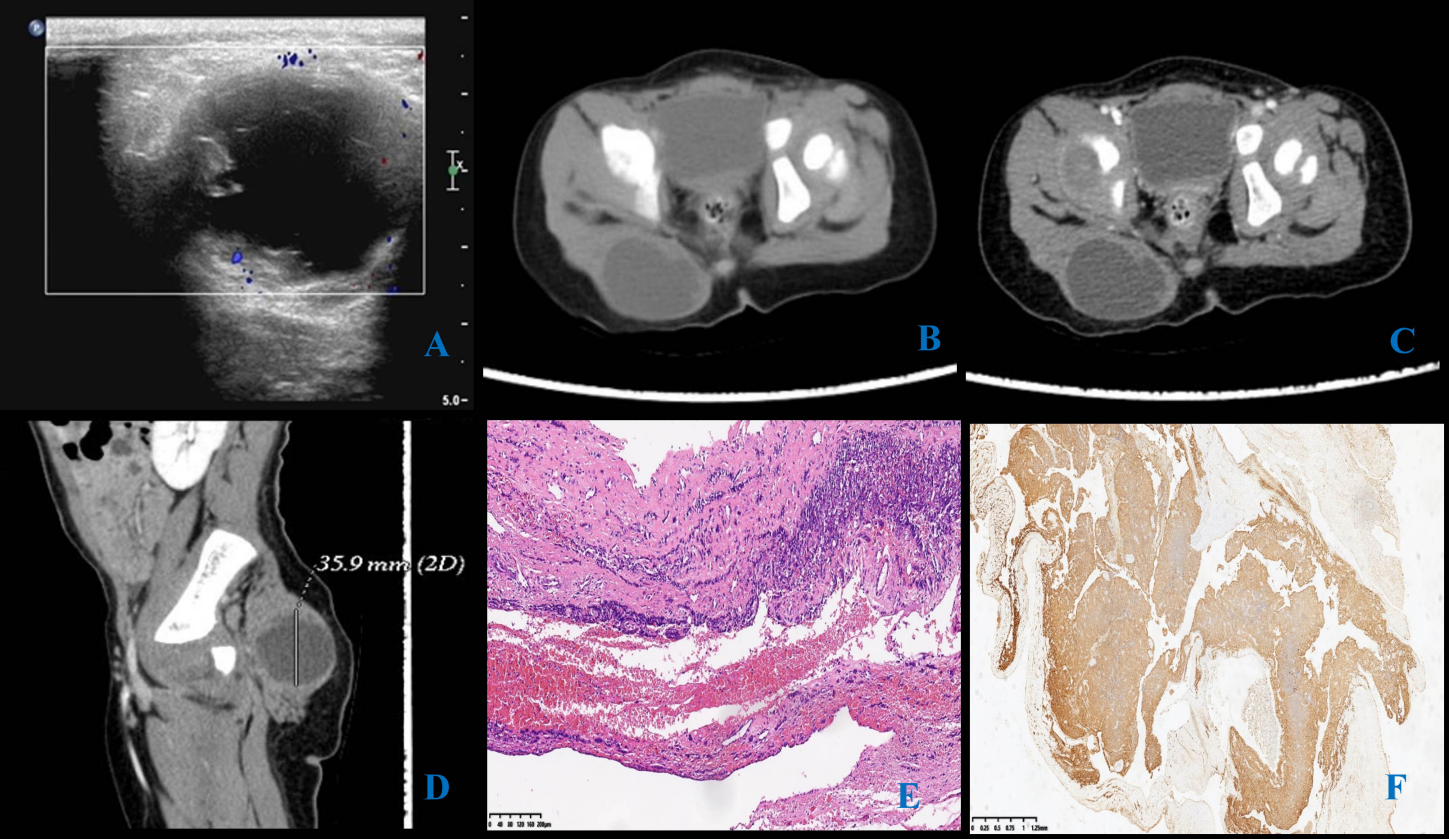
Ultrasound and CT images of the mass. **A**). Ultrasound image of the mass; **B**-**D**). CT images of the mass; **E**). HE staining (X200); **F**). CD99 immunohistochemistry: diffusively expressed in the tumor cell membrane.

A cystic mass was identified in the soft tissue of the right buttock, initially suspected to be a lymphatic malformation. Color Doppler Flow Imaging (CDFI) showed no significant blood flow signals (Fig. **1A**).

#### 2.1.3 CT Scan

A cystic mass with a thick wall was noted under the skin of the right buttock. Following contrast administration, slight enhancement was noted at the mass’s edges, with no enhancement or contrast agent uptake observed within the mass itself. The boundary remained clear, measuring approximately 44.5 mm×35.9 mm×30.3 mm (Fig. **1B-D**).

#### 2.1.4 Preoperative Diagnosis

The mass was initially considered to be a lymphatic malformation, and surgical resection was performed.

#### 2.1.5 Pathology

Postoperative pathology indicated that the tumor lacked an obvious capsule. The tumor cells were round, with sparse cytoplasm, lightly stained, and exhibited vacuolated nuclei with few mitotic figures. A large number of vascular structures and blood vessel invasions were observed around the tumor (Fig. **1F**). Fluorescence *in situ* hybridization (FISH) showed a positive EWSR1 gene break, and immunohistochemistry showed diffuse CD99 expression on the tumor cell membrane (Fig. **1E**), leading to a diagnosis of ES.

#### 2.1.6 Treatment

Postoperative chemotherapy with the PNET Phase II CAV/IE regimen was administered, along with radiotherapy. After 1 year and 6 months, pulmonary nodules were noted, suggesting pulmonary metastasis. Surgical resection of the lung nodules was performed, followed by continued chemotherapy.

### 2.2 CASE 2

A 15-year-old girl was admitted to the hospital after noticing a mass in the back of her neck for one day. The patient had no other symptoms and was in good general condition.

#### 2.2.1 Physical Examination

A protruding mass was observed at the back of the neck with normal skin surface and no ulceration. The mass was soft in texture and non-tender to palpation.

#### 2.2.2 Ultrasound

**Fig. (2):**
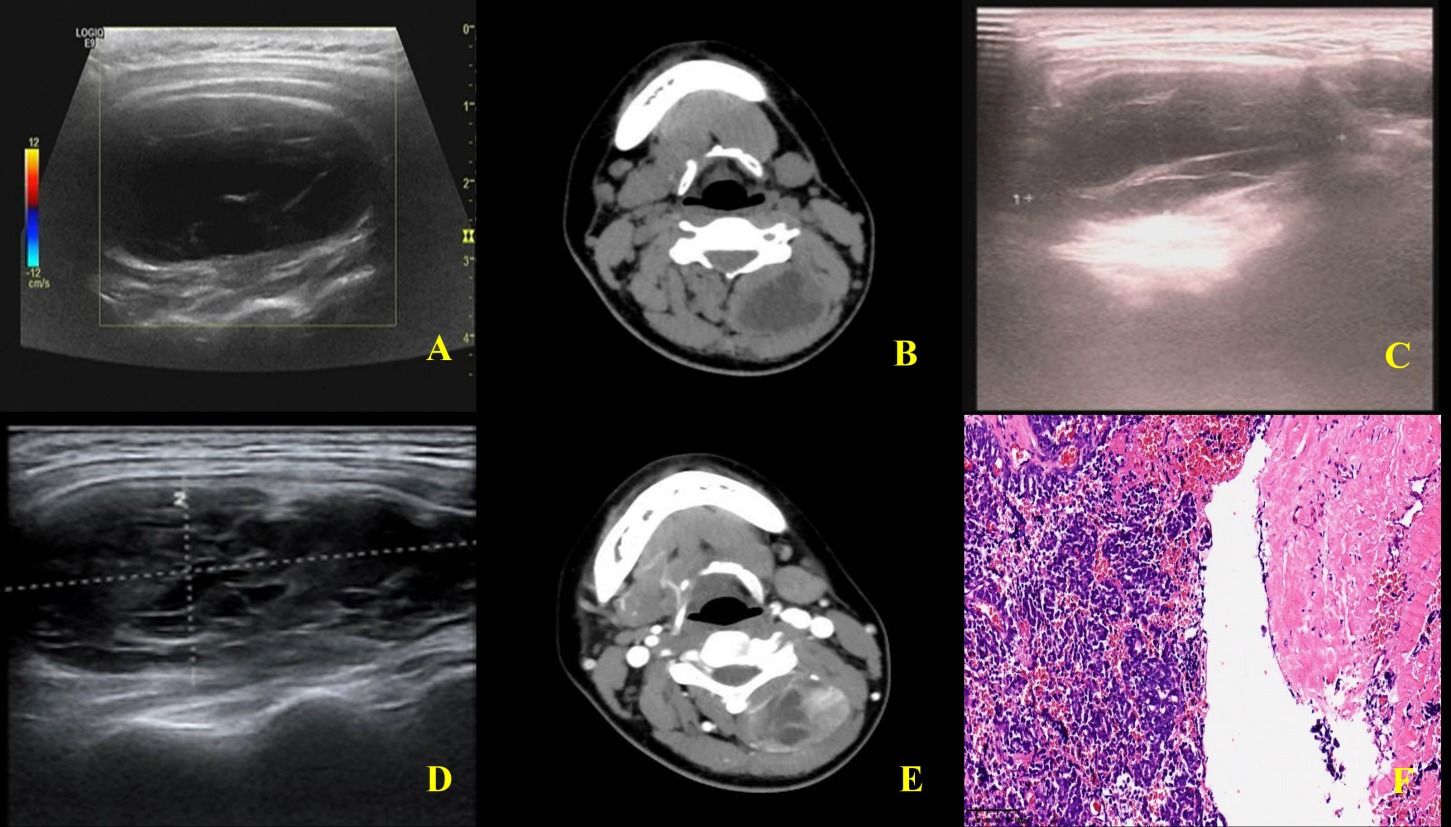
Imaging images and pathology of the mass. **A**). Ultrasound image before treatment, **B**). CT image before treatment, **C**). Ultrasound image 1 month after treatment, **D**). Ultrasound image 2 months after treatment, **E**). CT image 2 months after treatment, **F**). HE staining of the mass (X200): flaky tumor cells and flaky bleeding were found in the cyst cavity.

A cystic mass with clear boundaries, measuring approximately 49mm*35mm*25mm, was seen in the deep layer of the muscles at the back of the neck. Internal septations were visible, and no blood flow signals were detected within the mass (Fig. **2A**).

#### 2.2.3 Neck CT Scan

A cystic mass was observed in the left posterior cervical muscle space at the level of C3-C5 vertebrae. The mass was initially suspected to be a lymphangioma (Fig. **2B**).

#### 2.2.4 Treatment

Due to the family's concerns about the trauma of surgery, an intralesional injection of pingyangmycin was administered. During the procedure, 15 mL of pale, bloody fluid was aspirated. One month postoperatively, a follow-up neck ultrasound showed a reduction in size to approximately 38mm×34mm×20mm (Fig. **2C**). A second intralesional injection of pingyangmycin was performed, with 5 mL of pale bloody fluid aspirated.

#### 2.2.5 Follow-up and Progression

One month after the second injection, a follow-up ultrasound showed no significant change. Two months later, the ultrasound suggested an increase in size to approximately 59mm×45mm×26mm (Fig. **2D**). A CT scan indicated a slightly mixed low-density shadow in the left posterior cervical muscle space at the level of C3-C5 vertebrae, with unclear boundaries with adjacent muscles and obvious enhancement (Fig. **2E**).

#### 2.2.6 Surgical Intervention

Surgical resection of the neck mass was performed. During the procedure, the mass was found to be closely adherent to the anterior and posterior walls of the head and neck muscles, including the splenius capitis, semispinalis capitis, and longissimus capitis muscles. The tumor was completely resected.

#### 2.2.7 Pathology and Diagnosis

Intraoperative frozen pathology suggested a small round-cell tumor, prompting an extended resection. The diagnosis of ES was made based on routine pathology, immunohisto-chemistry, and genetic testing (Fig. **2F**).

#### 2.2.8 Postoperative Care

Postoperative chemotherapy using the PNET Phase II CAV/IE regimen was initiated. Follow-up to date showed no recurrence of the disease.

## 3 DISCUSSION

EES was first described by Tefft *et al.* in 1969 and named by Angervall in 1975. It accounts for 6%-47% of all ES cases [7], with head and neck EES representing about 2%-7% [8]. The incidence of EES is extremely low, with an average age of onset of 20 years and a slight male predominance. Both cases in this study were female, with case 1 being particularly rare at only 1 year and 1 month of age. EES lacks typical clinical manifestations, mainly presenting as a mass in the deep soft tissues. Early symptoms are often subtle, and some patients may experience mild local pain. In the two cases reported here, both masses were smaller than 5 cm and without local pain or other symptoms. Most EES patients can develop hematogenous metastasis early, commonly to the lungs, bones, or bone marrow, with local lymph node metastasis also a possibility [9]. Following the pathological diagnosis of ES in both cases, a comprehensive assessment of systemic lesions revealed no evidence of hematogenous metastasis or bone marrow involvement. Alan Alexander *et al.* [10] found that EES exhibits a wide range of imaging features, including areas of hemorrhage, necrosis, rich blood vessels, or partial cystic presence, and has low specificity for diagnosis.

Ultrasound imaging of EES typically shows a hypoechoic, heterogeneous solid mass in the soft tissue, with small echogenic stripes and mild internal blood flow [11, 12]. Ultrasound is useful in determining the cystic and solid nature of the lesion, assessing its morphology, size, capsule, relationship with surrounding tissues, and internal blood flow characteristics, aiding in preliminary assessment. The two cases reported in this study showed ultrasound findings of a cystic mass with clear boundaries located in the muscle interspaces, without involvement of the cervical vertebrae, and no obvious blood flow.

The CT manifestations of EES are non-specific, often showing unclear boundaries with surrounding tissues, heterogeneous enhancement, and low attenuation areas corresponding to hemorrhage or necrosis [13]. Zeyang Chen *et al.* [14] summarized the CT imaging characteristics of 25 intra-abdominal ES cases and found that primary EES often showed lobulated contours (87.5%), no calcification (75%), severe necrosis or cystic degeneration (75%), non-uniform enhancement (100%), moderate enhancement (75%), unclear boundaries (62.5%), and organ invasion (75%). The large size and rapid growth of EES, combined with insufficient blood supply, often lead to ischemic necrosis within the tumor [15, 16]. However, the CT in this report showed a cystic mass with irregular inner edges and thick walls, suggesting that the mass had boundaries distinguishable from surrounding tissues, differing from typical EES features, leading to potential misdiagnosis.

MRI is also non-specific in assessing EES. Soft tissue masses typically show similar or lower signals compared to skeletal muscle on T1-weighted images (T1WI) and medium to high signals on T2-weighted images (T2WI). Hemorrhagic areas show high signals on all sequences, while necrotic areas manifest as low signals on T1WI and high signals on T2WI [4]. Due to the higher cost, MRI is less commonly used as an initial examination in underdeveloped areas, often being conducted after a CT scan.

Under light microscopy, ES tumor cells are uniformly small round cells with round or oval nuclei, scant cytoplasm, and lightly eosinophilic staining. The diagnosis is confirmed by the EWSR1 gene rearrangement. Although lymphatic components are rarely a CT feature in EES [17], the pathological findings in these cases revealed abundant vascular tissue, consistent with previously studied CT features and possibly contributing to the cystic component presentation.

Differential diagnoses for ES include synovial sarcoma, rhabdomyosarcoma, neuroblastoma, and lymphoma, with differentiation relying on immunohistochemistry and detection of characteristic chromosomal translocations. Additionally, differentiation is needed from benign solid tumors, such as lymphatic vascular malformations, soft tissue abscesses, and hematomas [18]. Both patients in this study were initially misdiagnosed with lymphatic malformations.

A comprehensive treatment approach combining surgery and chemoradiotherapy is recommended for EES, as it has been shown to improve the 5-year survival rate [19, 20]. While radiotherapy plays a key role, studies have found that adding radiotherapy to local EES patients who have undergone chemotherapy and surgical resection does not improve overall survival. It may lead to radiation-related complications, including muscle atrophy, pathological fractures, and secondary malignant tumors.

## CONCLUSION

The early diagnosis of EES is challenging. For infants, children, or adolescents presenting with palpable masses, ultrasound is the primary examination method. However, both CT and MRI have limitations in specificity. There are no specific tumor markers for ES. When a cystic lesion is found in the muscle interstitial space, and surgery reveals fish-flesh-like tissue, or if the lesion does not improve after conservative treatment, EES should be considered as a potential diagnosis. Prompt recognition is crucial to avoid misdiagnosis or missed diagnosis, ensuring appropriate management and treatment.

## Data Availability

The data supporting the findings of the article are available within the article.

## LIST OF ABBREVIATIONS

CT: Computed Tomography
ES: Ewing Sarcoma

## STANDARDS OF REPORTING

CARE guidelines were followed.
